# A Phase I Dose-Escalation Trial of Single-Fraction Stereotactic Radiation Therapy for Liver Metastases

**DOI:** 10.1245/s10434-015-4579-z

**Published:** 2015-05-12

**Authors:** Jeffrey J. Meyer, Ryan D. Foster, Naama Lev-Cohain, Takeshi Yokoo, Ying Dong, Roderich E. Schwarz, William Rule, Jing Tian, Yang Xie, Raquibul Hannan, Lucien Nedzi, Timothy Solberg, Robert Timmerman

**Affiliations:** Department of Radiation Oncology, UT Southwestern Medical Center, Dallas, TX USA; Department of Radiology, UT Southwestern Medical Center, Dallas, TX USA; Department of Surgery, Indiana University Health Goshen Center for Cancer Care, Indiana University School of Medicine, Goshen, IN USA; Department of Radiation Oncology, Mayo Clinic Arizona, Phoenix, AZ USA; Department of Clinical Sciences, UT Southwestern Medical Center, Dallas, TX USA; Department of Radiation Oncology, University of Pennsylvania, Philadelphia, PA USA

## Abstract

**Background:**

There is significant interest in the use of stereotactic ablative radiotherapy (SABR) as a treatment modality for liver metastases. A variety of SABR fractionation schemes are in clinical use. We conducted a phase I dose-escalation study to determine the maximum tolerated dose of single-fraction liver SABR.

**Methods:**

Patients with liver metastases from solid tumors, for whom a critical volume dose constraint could be met, were treated with single-fraction SABR. Seven patients were enrolled to the first group, with a prescription dose of 35 Gy. Dose was then escalated to 40 Gy in a single fraction, and seven more patients were treated at this dose level. Patients were followed for toxicity and underwent serial imaging to assess lesion response and local control.

**Results:**

Fourteen patients with 17 liver metastases were treated. There were no dose-limiting toxicities observed at either dose level. Nine of the 13 lesions assessable for treatment response showed a complete radiographic response to treatment; the remainder showed partial response. Local control of irradiated lesions was 100 % at a median imaging follow-up of 2.5 years. Two-year overall survival for all patients was 78 %.

**Conclusions:**

For selected patients with liver metastases, single-fraction SABR at doses of 35 and 40 Gy is tolerable and shows promising signs of efficacy at intermediate follow-up.

Dissemination to the liver is a common event in the metastatic progression of many types of tumors. In a subset of patients, local therapies, such as surgery directed to metastatic lesions growing in the liver, may lead to prolonged disease-free survivals beyond what would be expected with systemic therapy alone.^1^,^2^ For the treatment of colorectal cancer, it is well established that complete surgical extirpation of hepatic metastases can yield long-term disease-free survival in a significant proportion of selected patients. In patients with oligometastatic disease states where the metastatic burden is limited to the liver, removal or ablation of these tumors may lead to cure.^3^

Surgical intervention is limited to a selected group of patients who are candidates based on medical operability and also have resectable disease with sufficient hepatic reserve. As a result, there is a growing variety of minimally invasive treatment alternatives to resection for the management of liver metastases. These treatments have primarily taken the form of thermal ablation, most commonly radiofrequency ablation (RFA). RFA has yielded good local control results, in particular for smaller (<3 cm) tumors removed from large blood vessels where the heat-sink effect limits ablative temperatures.^4^^,^^5^

Recent developments in radiation treatment planning techniques and technologies have allowed for the application of stereotactic ablative radiotherapy (SABR), which delivers potent radiation doses to discrete tumors with rapid fall-off of dose in surrounding normal tissues, to targets in the lung, liver, and other organs. SABR delivered in one fraction (one treatment) is a promising approach for many reasons. Single-fraction treatment is highly convenient for patients and efficient, leading to minimal interruption in planned systemic therapies for patients with metastatic cancer. Herfarth and colleagues reported an early experience with single-fraction SABR, escalating the prescription dose from 14 to 26 Gy with promising safety and early efficacy results.^6^ Authors from the same institution recently reported a series of 138 liver tumors in 90 patients treated with single-fraction therapy, with prescription doses as high as 30 Gy.^7^ With a median dose of 24 Gy, local control at 18 months was 59 %. Patients with colorectal primary tumors had worse local control rates relative to breast primary tumors. Goodman and colleagues safely escalated single-fraction irradiation from 18 to 30 Gy for patients with primary and secondary liver tumors.^8^ Local control was 77 % at 1 year.

These series imply that higher radiation doses may be necessary for optimal tumor control. However, the safety and tolerability of higher-dose single-fraction radiation treatments for liver tumors is unclear. Therefore, we conducted a phase I clinical dose-escalation trial to determine the tolerability of single-fraction SABR in the liver. In this report, we detail treatment-related toxicities of this approach as well as treatment efficacy results.

## Methods

### Patient Eligibility

This phase I clinical trial was approved by the UT Southwestern institutional review board. Adult patients with Zubrod performance status of 2 or less, with 5 or fewer liver metastases (non-germ cell or hematologic origin) were eligible for enrollment. Patients had to have an expected life span of at least 6 months and could not have received prior liver radiation, which would lead to exceeding protocol-defined constraints for the liver and other normal tissues. Patients underwent multidisciplinary evaluation for consideration of liver-directed therapies. The treated tumor(s) had to be located outside of the central liver zone, defined as a 2-cm expansion around the course of the portal vein contoured to its bifurcation in the liver. Patients were not eligible if they had significant and uncontrolled active comorbidities, including any or all of the following: unstable angina and/or congestive heart failure or myocardial infarction within the preceding 6 months; COPD exacerbation or acute infection at the time of registration; or active hepatitis or Child’s-Pugh class B or C cirrhosis. Patients’ laboratory values had to meet these restrictions: hemoglobin ≥10 g/dL, Platelets ≥100,000 per microliter, ANC ≥1000 per microliter; albumin >3 g/dL, alkaline phosphatase, ALT, AST, total bilirubin, and PT/INR ≤1.5 times the upper limit of normal. A critical liver volume constraint also had to be met with the single-fraction radiation treatment plan: at least 700 cc of normal liver had to receive <9.1 Gy. Steroid premedication was encouraged.

### Radiation Dose Escalation

The radiation prescription dose was escalated in increments of 5 Gy. The starting dose was 35 Gy. Dose-limiting toxicity (DLT) was defined according to the National Cancer Institute Common Toxicity Criteria for Adverse Events (CTCAE). Treatment-related grade 3, 4, or 5 gastrointestinal, hepatobiliary/pancreatic, renal/genitourinary, or neurologic toxicity was considered dose-limiting toxicity, as were any grade 4 or 5 toxic events in these categories: blood/bone marrow, cardiac, pulmonary/upper respiratory, metabolic/laboratory, musculoskeletal/soft tissue, and skin. Any other grade 4 or 5 events that were treatment-related also constituted DLT.

Patients were enrolled in cohorts of 7–15. If zero of the first 7 patients, 2 or fewer of the first 9, 3 or fewer of the first 12, or 4 or fewer of the first 15 experienced DLT within the first 90 days following treatment, the dose level was escalated. The maximum tolerated dose level was established as the tolerated dose level below which excessive DLTs (at a rate ≥33 %) had been reached.

### Single-Fraction SABR Planning and Delivery

Patients were immobilized in a full-body, vacuum-lock mold, which was placed in a stereotactic body frame (Elekta, Stockholm, Sweden). Respiratory motion was assessed with fluoroscopy and motion of the diaphragm was limited to <1 cm with the use of abdominal compression as needed. Placement of fiducials to assess motion and guide radiation delivery was allowed but not required. A four-dimensional computed tomography (CT) scan was obtained to aid in delineation of the motion envelope of the gross tumor volume. A magnetic resonance imaging (MRI) scan of the abdomen, unless contraindicated, was recommended to aid in delineation of the tumor for treatment planning. This scan could serve as a baseline image set for assessment of treatment response. Expansions of 5 mm in the axial plane and 5–10 mm in the cranio-caudal plane around the GTV were made to generate a planning target volume (PTV). Both three-dimensional (3D) conformal radiation treatments and intensity-modulated radiation therapy with megavoltage photons could be used for treatment planning. For the 3D conformal treatments, multiple noncoplanar beams were used, with minimal block margin surrounding the PTV. As such, prescription isodose lines for these plans could be to the 60–90 % isodose line. Planning guidelines called for 95 % of the PTV to be covered by the prescription dose and 99 % of the PTV to receive a minimum of 90 % of the prescription dose. Normal tissue dose delivery guidelines for bowel, skin, lung, and kidney were provided to facilitate planning but could be exceeded at the discretion of the treating physician. The liver critical volume and spinal cord constraints had to be respected in all cases. Normal tissue constraints are shown in Table 1.

**Table 1:** Normal tissue dose constraints

Structure	Constraint
Uninvolved liver	700 mL receives <9.1 Gy
Spinal cord	<0.35 mL exceeds 10 Gy
	<1.2 mL exceeds 7 Gy
	Maximum allowed point dose^a^: 14 Gy
Stomach	<10 mL exceeds 11.2 Gy
	Maximum allowed point dose: 12.4 Gy
Duodenum	<5 mL exceeds 11.2 Gy
	<10 mL exceeds 9 Gy
	Maximum allowed point dose: 12.4 Gy
Jejunum/ileum	<5 mL exceed 11.9 Gy
	Maximum allowed point dose: 15.4 Gy
Colon	<20 mL exceed 14.3 Gy
	Maximum allowed point dose: 18.4 Gy
Skin	<10 mL exceed 23 Gy
	Maximum allowed point dose: 26 Gy

^a^Point dose = 0.035 mL

### Follow-up

Patients were followed at 4–6 weeks and at 3 months following treatment, with subsequent follow-up visits every 3 months through 1 year. Then, patients were followed every 6 months through 5 years of follow-up, and annually thereafter. At the time of follow-up, imaging of the abdomen, preferably with MRI, was obtained, as were the following: serum chemistries and PT/INR.

Local control was defined as lack of tumor growth on imaging studies within the treated tumor volume. Pre- and post-SABR MRI scans were reviewed to assess control on a per-lesion basis. T2-weighted axial images were followed as these images most consistently and clearly delineated the target tumor for radiation treatment planning. Follow-up and survival were calculated from the day of radiation treatment.

Treatment with systemic therapy was allowed both before and after the single-fraction SABR treatment. However, it had to be held for 6 days before, during, and 5 days after the delivery of the radiation.

### Primary and Secondary End Points

The primary end point of this study was to establish a maximum tolerated dose for single-fraction irradiation of liver metastases. The initial goal was to escalate to a dose of 50 Gy if dose-limiting toxicities were not prohibitive at lower doses. However, with feasibility issues, including patient accrual as well as the local control results obtained at 40 Gy, further escalation beyond 40 Gy was not undertaken. Secondary endpoints included local control of the treated lesions and overall survival.

## Results

### Patient Characteristics

Patient and tumor characteristics are shown in Table 2. Fourteen patients with 17 liver metastases were treated: seven at the first dose level (35 Gy) and seven at the second level (40 Gy). Patients had a variety of primary tumor sites. Three patients had two liver metastases treated. None of the tumors abutted the inferior vena cava. One treated tumor abutted the left hepatic vein.

**Table 2:** Patient and tumor features

Characteristic	Value
Number of patients	
Dose level 1 (35 Gy)	7
Dose level 2 (40 Gy)	7
Age (yr)	
Median	61
Range	39–82
Sex	
Male	9 (64.3 %)
Female	5 (35.7 %)
Primary site	
Renal	5 (35.7 %)
Colorectal	3 (21.4 %)
Melanoma	2 (14.3 %)
Nasopharynx	1 (7.1 %)
Lung	1 (7.1 %)
Breast	1 (7.1 %)
Endometrial	1 (7.1 %)
Number of tumors	17
Tumors treated per patient	
1	11
2	3
PTV volume (mL)	
Median	27.95
Range	4.08–79.34

### Toxicity

There were no treatment-related grade 3, 4, or 5 events at either dose level. A patient in the 40-Gy group developed grade 2 nausea and vomiting as well as a grade 2 increase of alkaline phosphatase, all of which were considered related to the radiation treatment. All other patients had at worst grade 1 toxicity events (toxicity outcomes are shown in Table 3). Thus, there were no DLT events as defined by the protocol. Four patients developed postradiation biliary stenosis adjacent (peripheral) to the treated tumor: two patients in each dose group. In two cases this was segmental stenosis, and in two cases it was subsegmental. No intervention was required in any of these cases. Four patients with tumors near the liver dome developed asymptomatic imaging changes in the lung adjacent to the dome.

**Table 3:** Toxicities

Toxicity	Grade 1	Grade 2
Fatigue	4	0
Alkaline phosphatase increased	5	1
Abdominal pain	1	0
Creatinine increased	1	0
Chills	2	0
Nausea	2	1
Vomiting	1	1
Fever	1	0
Aspartate aminotransferase increased	2	0
Alanine aminotransferase increased	2	0
Chest wall pain	2	0
Bile duct stenosis	4	0

^a^All toxicities deemed at least possibly related to the stereotactic radiation treatment. Some patients had multiple toxicities and these are separately documented in this table

### Tumor Response and Local Control

**Fig. 1:**
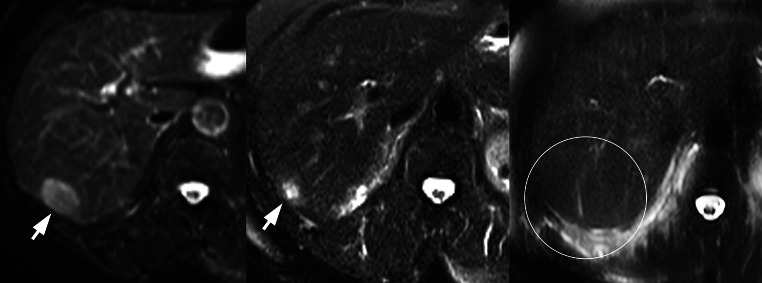
Radiographic response of a treated liver metastasis. The patient had a diagnosis of metastatic non-small cell lung cancer with evidence for a liver metastasis in segment 6. The *left panel* shows the pretreatment T2 fat-saturated image with the lesion noted by the *arrow*. The *central panel* shows the treated lesion approximately 6 weeks following radiation (35 Gy). The *right panel* shows the lesion approximately 6 months following radiation, with no evidence of residual tumor

Eleven of the 14 patients had baseline MRI scans obtained before the SABR treatment, and all patients had MRI obtained at follow-up visits as per the protocol guidelines. Applying RECIST criteria in the MRI-based assessment of irradiated liver metastases is challenging, because there are multiple sequences obtained with MRI.^9^ We assessed response based on consistent imaging sequences (T2) across the studies. Using this criteria, and based on the 13 treated metastases, which were ≥1 cm on baseline imaging and which had both pre- and posttreatment MR assessment, the median imaging follow-up time was 2.5 (range 0.5–3.0) years. None of these 13 tumors had evidence for local progression at the time of last follow-up; thus, treated lesion local control was 100 %. Nine of these 13 (69 %) tumors demonstrated a complete response, and 4 of these 13 (31 %) tumors demonstrated a partial response at the time of last imaging follow-up. Figure 1 demonstrates the imaging evolution of one of the liver metastases treated in this study.

Allowing for CT-to-MRI comparisons as well as comparison of lesions <1 cm in size, the median imaging follow-up for all 17 of the treated lesions was 2.5 (range 0.5–3.5) years. In this analysis, local control was again 100 % with no tumor showing local progression at the time of last follow-up

### Survival

**Fig. 2:**
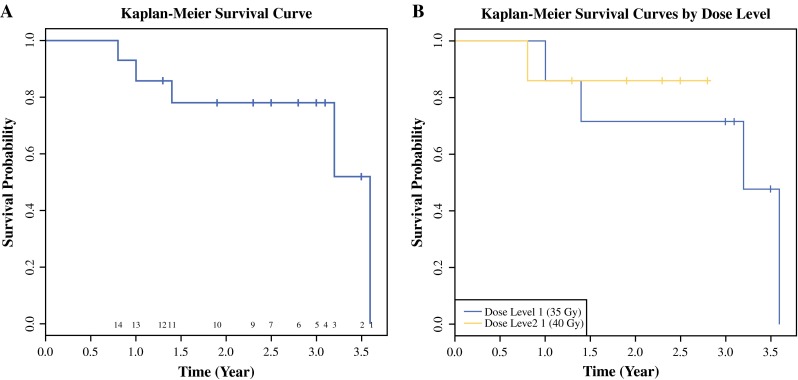
**a** Overall survival curve for all treated patients. **b** Overall survival curve for the two dose groups

Nine of 14 treated patients were still alive at the time of survival analysis. Figure 2 shows Kaplan–Meier survival curves for all 14 patients and patients grouped by dose levels. Five of the 14 treated patients have died. None of the deaths were deemed treatment-related. Estimated 2-year overall survival was 78 %.

## Discussion

A variety of ablation technologies are available to treat tumors, including foci of metastatic disease, as alternatives to surgery. SABR is increasingly well established as a treatment therapy for tumors in the lung, liver, prostate, kidney, bone, and other organs.^8^^,^^10^–^22^ Liver tumors were one of the first types of neoplasm treated with SABR. Blomgren and colleagues reported an early series with different hypofractionated regimens in the mid-1990s.^23^ Subsequently, there have been clinical investigations into various high-dose hypofractionation regimens.^8^,^11^–^17^

Because the liver has features of a parallel-structured organ, at least in its periphery, tolerance to partial volume liver irradiation is substantially higher relative to whole-volume irradiation.^24^ Also, it is established that a limitation imposed on surgery for liver metastases relates to the requirement to leave a remnant of liver parenchyma that is sufficient to maintain immediate hepatic function.^25^ In our study, we used a baseline “critical volume” of liver thought necessary to maintain baseline liver function. In addition, a tolerance dose, in essence a threshold dose, was defined above which liver parenchyma would be significantly damaged. Liver parenchyma exposed to doses below the tolerance dose could contribute to the critical volume and would be assumed to maintain essential hepatic function after the treatment. This same model tested in our current trial’s design allowed high levels of dose escalation in previous phase I trials for SABR treatment of liver metastases using 3 and 5 fractions.^11^,^12^

High-dose radiation treatments, including single-fraction SABR, may eradicate tumors through stromal effects not predicted by classical radiation biology considerations.^26^–^28^ Investigators from Memorial Sloan-Kettering have reported significantly improved local control for doses in the 23–24 Gy range relative to lower doses.^29^–^32^ It is possible that doses higher than this are required as a function of tumor histology and location, because doses in the range of >20 Gy have not always been associated with high and durable local tumor control rates in other series. Thus, phase I dose escalation studies such as our own are warranted.

In our study, we escalated the radiation dose from 35 to 40 Gy in a single fraction for tumors outside of the central liver zone, which we defined as a 2-cm shell surrounding the portal vein to its bifurcation in the liver. We chose this selection criteria based on concern that the liver may demonstrate features of a radiation-sensitive, serial-structured organ near its hilum. Prior experience in treatment of central lung tumors (lesions near the major mainstem bronchi) demonstrated excessive toxicity for severely hypofractionated treatment courses relative to what was seen with tumors in the lung periphery.^33^ Therefore, it seemed prudent to exclude central liver tumors from high-dose single-fraction treatments, although a separate clinical study, or possibly preclinical study, would be necessary to determine if the structures in the hilum of the liver exhibit sensitivity similar to the lung hilum.

We used serial MR imaging to follow the response of treated lesions and observed promising local control results at both dose levels. The response of both tumors and normal liver on MRI to high- and intermediate-dose radiation is not well described in the literature, and application of RECIST criteria to tumors followed with MR imaging is challenging because of the multitude of sequences obtained with scans. We used consistent sequences (T2) to follow patients from baseline through serial follow-up. Further follow-up and study will help to determine if the excellent local control rates are sustained and will aid in determining if stereotypical imaging responses of tumor and normal tissue following high-dose radiation are observed and correlated with long-term tumor control and normal liver injury.

## Conclusions

With the selection criteria used in our study design, we were able to safely escalate single fraction SABR for liver metastases to 40 Gy. Based on these results, we propose that this is a safe and apparently effective alternative to multi-fraction SABR courses for well-selected patients with liver metastases.
